# Celecoxib concentration predicts decrease in prostaglandin E_2 _concentrations in nipple aspirate fluid from high risk women

**DOI:** 10.1186/1471-2407-8-49

**Published:** 2008-02-11

**Authors:** Edward R Sauter, Wenyi Qin, John E Hewett, Rachel L Ruhlen, John T Flynn, George Rottinghaus, Yin-Chieh Chen

**Affiliations:** 1 Department of Surgery, University of Missouri, Columbia, MO, USA; 2 Department of Biostatistics, University of Missouri, Columbia, MO, USA; 3 Department of Physiology, Thomas Jefferson University, Philadelphia, PA USA; 4 The Veterinary Medical Diagnostic Laboratory, University of Missouri-Columbia, Columbia, MO USA

## Abstract

**Background:**

Epidemiologic studies suggest that long term low dose celecoxib use significantly lowers breast cancer risk. We previously demonstrated that 400 mg celecoxib taken twice daily for 2 weeks lowered circulating plasma and breast nipple aspirate fluid (NAF) prostaglandin (PG)E_2 _concentrations in post- but not premenopausal high risk women. We hypothesized that circulating concentrations of celecoxib influenced PGE_2 _response, and that plasma levels of the drug are influenced by menopausal status. To address these hypotheses, the aims of the study were to determine: 1) if circulating plasma concentrations of celecoxib correlated with the change in plasma or NAF PGE_2 _concentrations from baseline to end of treatment, and 2) whether menopausal status influenced circulating levels of celecoxib.

**Methods:**

Matched NAF and plasma were collected from 46 high risk women who were administered celecoxib twice daily for two weeks, 20 subjects receiving 200 mg and 26 subjects 400 mg of the agent. NAF and plasma samples were collected before and 2 weeks after taking celecoxib.

**Results:**

In women taking 400 mg bid celecoxib, plasma concentrations of the agent correlated inversely with the change in NAF PGE_2 _levels from pre- to posttreatment. Nonsignificant trends toward higher celecoxib levels were observed in post- compared to premenopausal women. There was a significant decrease in NAF but not plasma PGE_2 _concentrations in postmenopausal women who took 400 mg celecoxib (p = 0.03).

**Conclusion:**

In high risk women taking 400 mg celecoxib twice daily, plasma concentrations of celecoxib correlated with downregulation of PGE_2 _production by breast tissue. Strategies synergistic with celecoxib to downregulate PGE_2 _are of interest, in order to minimize the celecoxib dose required to have an effect.

## Background

Cyclooxygenases (COX)-1 and COX-2 may be present in breast tumors to catalyze the conversion of arachidonic acid to prostaglandins, prostacyclins, or thromboxanes. While COX-1 expression is constitutive, COX-2 is inducible [1] and is upregulated in a variety of tumors, including breast cancers [2]. Prostaglandin (PG)E_2_, which has tumor and cell growth promoting activity [3], is produced from arachidonic acid by either COX-1 or -2. Malignant breast tumors produce more PGE_2 _than benign breast tumors or normal breast tissue [2]. Women with breast cancer with TUMOR PGE_2 _levels above 15 ng/g appear to have a significantly worse survival rate than those with levels ≤ 15 ng/g [4].

Nonsteroidal antiinflammatory drugs (NSAIDs), including aspirin, indomethacin and ibuprofen, inhibit both COX-1 and COX-2. Inhibition of COX-1 leads to a number of adverse effects, including gastrointestinal ulcers and renal toxicity [5]. Recent efforts have therefore focused on pharmacologic agents such as celecoxib, a clinically available medication which selectively inhibits COX-2. Preclinical and epidemiologic studies suggest that celecoxib is effective both in preventing and in treating breast cancer in a dose dependant manner [6-9]. A case control study of women with and without breast cancer who were evaluated during a 12 month period found that women who had used 200 mg celecoxib daily for 2 years or longer were 83% less likely to be diagnosed with breast cancer than were controls [8]. Two international, multicenter studies using celecoxib to prevent sporadic colorectal adenomas were recently reported, the Adenoma Prevention with Celecoxib (APC) and the Prevention of Colorectal Sporadic Adenomatous Polyps (PreSAP) trials. Both demonstrated the efficacy of celecoxib in preventing colorectal adenomas after three years of treatment. Compared to placebo, the APC, but not PreSAP study, demonstrated an increased risk of cardiovascular events in the celecoxib arm. The APC study administered celecoxib 200 or 400 mg bid, whereas the PreSAP study was a single daily 400 mg dose. Mean plasma concentrations of celecoxib were not measured in either study.

Circulating celecoxib concentrations have been measured in preclinical treatment and in pharmacokinetic human trials. Improved survival of mice with erythroleukemia was demonstrated by a combination of low dose vincristine [10] in combination with a dose of celecoxib which provided average circulating celecoxib concentrations of 2198 ng/mL (5.77 μM). Circulating concentrations of 876 ng/mL (2.3 μM) celecoxib slowed the growth of HCA-7 human colorectal cancer xenografts in nude mice [11]. The half life of celecoxib averages 11 hours in fasting patients [12]. Adminstration of 400 mg celecoxib daily to 68 healthy adults for 2 weeks, with the time from last dose to blood collection ranging from 9–24 hrs, led to a mean plasma concentration of 607 +/- 338 ng/ml (range 82–1700 ng/mL) [13].

We are currently able to collect breast nipple aspirate fluid (NAF) from 95% of nonlactating adult females with the use of a modified breast pump [14]. Median PGE_2 _concentrations in NAF were 55 times higher than in matched plasma prior to treatment with celecoxib [15]. We found that 400 mg twice daily (bid) of the COX-2 inhibitor celecoxib administered for two weeks to women at increased breast cancer risk significantly decreased PGE_2 _levels in the breast, as measured both in NAF and in plasma [16]. We sought to extend these findings to determine: 1) if circulating concentrations of celecoxib correlated with change in plasma or NAF PGE_2 _from baseline to end of treatment, and 2) whether menopausal status influenced circulating concentrations of celecoxib. We observed that the circulating plasma concentration of celecoxib was related to the reduction in PGE_2 _in NAF but not in plasma at the end of celecoxib therapy in high risk women receiving 400 mg celecoxib twice daily.

## Methods

### Subject recruitment

Women were provided an Institutional Review Board approved protocol and required to give written informed consent in order to enroll in the study. Subjects evaluated had to be ≥ 18 years old and be at increased breast cancer risk, based on the subject having either a Gail model risk of developing invasive breast cancer (IBC) in a 5 year period of > 1.66%, or previously treated ductal carcinoma *in situ *(DCIS) or IBC (now finished with treatment and free of disease).

Pregnant and lactating women were not eligible. Women could not have been currently on NSAIDs, aspirin, a COX-2 inhibitor, warfarin, or have taken such a medication within two weeks of enrollment. Subjects could not have a significant history of peptic ulcer disease, upper gastrointestinal bleeding, asthma, or be allergic to sulfonamides or NSAIDs. A complete blood count, serum electrolytes and liver panel had to be within normal limits. Subjects were recruited from the Breast Evaluation Clinics at the University of Missouri-Columbia.

### Intervention

Celecoxib pills were taken bid for 14 days. Compliance was assessed through the count of returned pills. All subjects were required to have taken at least 80% of the prescribed medication. The first 20 subjects recruited received 20 mg celecoxib twice daily. Analysis of the data from these subjects did not demonstrate a significant downregulation of pge2 in any subgroup. All subsequent subjects recruited received 40 mg celecoxib twice daily.

### Specimen collection

NAF was collected using a modified breast pump as previously described [14,17]. Briefly, the breast was warmed with moist heated towels for 5–10 minutes, subsequently massaged from the chest wall toward the nipple while a health care professional provided suction using a modified breast pump. The sample was collected into capillary tubes and stored at -80°c until analysis. NAF volume was measured using a metric ruler. We have determined that one mm in the tube corresponds to approximately one μL OF NAF. NAF was collected from only one breast, and NAF from the same breast was analyzed before and after treatment.

Baseline NAF and blood collection were performed prior to the ingestion of celecoxib. Eight mL of blood were also collected from the subject in a tube containing heparin, the blood spun for 10 min at 1600 rpm, and the plasma fraction decanted and stored at -80°C until analysis. All women had NAF and plasma collected within 24 hrs of their last dose of celecoxib, with an average of approximately 12 hrs. The half life of the medication is 11.5 hrs.

### Specimen analysis

#### PGE_2_

The biomarker chosen for analysis was PGE_2_, due to its established link to cancer growth. NAF and plasma samples were analyzed by immunoassay for their PGE_2 _content as per the manufacturer's instructions (R&D Systems, Minneapolis, MN). The kit uses a monoclonal antibody to PGE_2 _to competitively bind the PGE_2 _in the standard or sample. Briefly, samples were diluted in 100 μL assay buffer supplied by the manufacturer, pipetted into appropriate wells, incubated for 18–24 hrs at 4°C, washed, substrate solution added, followed by one hr incubation, and absorbance measured at 405 nm.

For NAF and plasma analyses, a standard curve was prepared using serial dilutions of PGE_2_. A linear regression equation was created from standards of known PGE_2 _concentration, and PGE_2 _concentrations of unknown samples fit to the standard curve regression equation, corrected for aliquot volume and expressed as nanograms of PGE_2_/mL of original sample. Whenever possible, NAF and plasma samples were run in duplicate and the average of the two values was reported. The goodness of fit of the standard curve, R^2^, for NAF samples was 0.999. The goodness of fit was similar for the plasma samples.

#### Celecoxib

Celecoxib was analyzed in plasma using a modification of the technique of Schonberger et al. [18] by combining 250 μL aliquots of plasma with an equal volume of distilled water and adding 500 uL ethanol to precipitate protein. Spiked plasma samples were prepared by combining 250 μL blank plasma with 250 μl distilled water, 20 μL of 10 ppm celecoxib in ethanol and 480 μL of ethanol. Samples were vortexed and then centrifuged at 13,000 rpm for 5 min. A 500 uL aliquot of the supernatant was combined with 1.5 mL distilled water and applied to a Waters 3 mL C_18 _Sep-Pac-Vac disposable cleanup column (Waters, Milford, MA) which was preconditioned with 2 mL methanol and then 2 mL distilled water. Cleanup columns were washed with 2 mL distilled water and vacuum dried for 15 minutes. Celecoxib was eluted with 4 mL methanol and the sample eluants taken to dryness. Samples were reconstituted in 1.0 mL methanol:water (80:20) for HPLC analysis.

HPLC analysis was performed on an Hitachi HPLC system (Hitachi Instruments, Inc., San Jose, CA) which consisted of an L7100 pump, with an L7200 autosampler (20 ul injected), and fluorescence detection with an L7480 fluorescence detector (excitation 240 nm, emission 380 nm). The system was controlled, data acquired and processed using an Hitachi D-7000 data acquisition package with Concert Chrom software on a microcomputer. A Phenomenex Hypersil BDS C_18 _analytical column (250 × 4.6 mm, 5 um) (Phenomenex, Torrence, CA) and a Phenomenex Securityguard C_18 _precolumn with a mobile phase of acetonitrile:water (70:30) was used at a flow rate of 1 mL/min. Celecoxib was kindly provided by Pfizer Corporation, New York, NY. A primary standard of celecoxib (2,000 ppm) was prepared in acetonitrile. Working standards (100, 200, 500 ppb) were prepared in methanol:water (80:20). Plasma samples spiked with celecoxib had recoveries greater than 95%.

Each sample batch that was run included a serum sample spiked with 200 ng/mL celecoxib to assess the recovery rate of the assay. The recovery rate was 99.5% +/- 3.4% (n = 11). In addition to assessing the recovery rate, we randomly selected 11 post treatment samples for duplicate analyses. We also analyzed six serum samples in duplicate that were collected before the subject started celecoxib. Each of the six baseline serum samples demonstrated zero values at both runs. The 11 post treatment samples each had measurable celecoxib levels, with the deviation within each set of these 11 samples having a CV < 10%.

### Toxicity

Among women taking celecoxib 200 mg bid, two subjects experienced side effects (one nausea, the second a cough), with both resolving spontaneously. There were no dropouts in the 200 mg bid group. Among women enrolled in the 400 mg bid group, 11 experienced side effects from celecoxib, four of whom dropped out. Of the four who dropped out, the side effects (edema in two, diarrhea in one, and heart palpitations in one) resolved shortly after stopping celecoxib. Among the remaining seven subjects, the side effects: diarrhea, nausea, rash, altered taste, urinary urgency, sweating, and muscle tension, all resolved spontaneously.

### Statistical analysis

Median values of continuous variables were computed for the various groups of subjects. Due to the potential non-normality of the data, ranking procedures were used for all analyses with continuous variables. The Wilcoxon Rank Sum Test was used to compare independent groups. Examples of these comparisons include comparing pre- and postmenopausal women, etc. The Wilcoxon Signed Ranks Test was used to make within group comparisons such as comparing pretreatment to posttreatment. Spearman's Correlation Coefficients were used to correlate quantitative variables such as age and celecoxib levels.

## Results

### Subjects

Between October 2001 and December 2004, informed consent was obtained from 54 women at increased breast cancer risk to enroll in an institutional review board approved protocol. Of the 54, 22 initiated celecoxib 200 mg bid and 32 celecoxib 400 mg bid. Two of the 54 (one in each dosage group) were not evaluable because plasma was not collected after treatment, preventing the measurement of celecoxib. Early in the study, two additional women (one in each dosage group) were excluded because we did not collect NAF from the same breast at baseline and after treatment. Later in the study we allowed the assessment of women who had plasma collected at both time points, even if matched NAF was not available, since we could still evaluate the association of circulating celecoxib concentrations with the systemic PGE_2 _response. Four additional subjects dropped out due to side effects, all in the 400 mg bid group, leaving 46 evaluable subjects. Side effects are discussed in greater detail below.

All 46 subjects provided NAF at their baseline visit. Reasons for not collecting NAF in 5 women at their follow-up visit included: one woman refused, in two women attempts to collect NAF were unsuccessful, and two women yielded less than one microliter of NAF, which we felt was insufficient for reliable analysis of PGE_2 _(Table 1). Plasma was collected in all subjects at all visits. In total, 41 matched NAF and 46 matched blood samples were collected at baseline and after two weeks of celecoxib treatment from 46 subjects.

**Table 1:** Demographics

	**Celecoxib Dose**	
	
	**200 mg twice daily**	**400 mg twice daily**
		
	**NAF (plasma)**	**NAF(plasma)**
**Samples**	19 (20)	22 (260
**Age (years)**		
Median	48 (47)	50.5 (50.5)
Range	23–68 (23–68)	30–81 (30–81)
**Premenopausal**	5 (6)	11 (11)
**Race**		
White	19 (20)	40 (45)
Black	0	0
Asian	0	0
American Indian	0	1 (1)
**Primary Risk Factors for Enrollment**		
Family History	11 (11)	14 (17)
Hyperplasia with/without atypia	3 (3)	6 (6)
History of Breast Cancer	3 (3)	3 (3)
History Prior Breast Biopsy	2 (3)	0

Half of the evaluable subjects in the 400 mg group, and 30% in the 200 mg group, were premenopausal. All but one subject recruited was Caucasian. In both dosage groups, the median number of celecoxib pills taken was over 98%, and all subjects took over 80% of the pills that they were given.

### Celecoxib concentrations in ng/mL

At the end of treatment, celecoxib was detectable in the plasma of 16 of 20 participants (80%) in the 200 mg group, and 20 of 26 participants (77%) in the 400 mg group. The limit of detection of the assay was 100 ng/mL. Of samples in which celecoxib was detectable, values ranged from 117.6 to 2281.2 ng/mL in the 200 mg group and from 156.8 to 16403.1 ng/mL in the 400 mg group. Levels trended (p = 0.08) higher in women taking 400 mg compared to 200 mg bid (Table 2).

**Table 2:** Median concentrations of PGE2 in NAF and celecoxib in plasma in ng/ml based on celecoxib dose^1^

**Population: Dose**		**Before (N)**	**After (N)**	**After v**.	**P value**	
		**Treatment**	**Treatment**	**Before(N)^2^**	**ΔPGE2**	**Celecoxib Level & ΔPGE_2_**
**200 mg twice daily**						
**Overall**	**PGE2**	13.75(19)	17.78(19)	0.37(19)	0.54	0.29
	**Celecoxib**		223.7(19)			
**Premenopausal**	**PGE2**	13.75(5)	21.85(5)	5.50(5)	0.63	
	**Celecoxib**		117.6(5)			
**Postmenopausal**	**PGE2**	14.15(14)	13.79(14)	-0.34(14)	0.86	
	**Celecoxib**		267.5(14)			
**400 mg twice daily**						
**Overall**	**PGE2**	13.80(22)	13.22(22)	-1.72(22)	0.83	**0.006**
	**Celecoxib**		759.8(22)			
**Premenopausal**	**PGE2**	15.7(11)	36.1(11)	7.31(11)	0.17	
	**Celecoxib**		227.3(11)			
**Postmenopausal**	**PGE2**	8.33(11)^3^	6.81(11)	-4.95(11)	**0.03**	
	**Celecoxib**		860.6(11)			

1: N: sample size; NAF: nipple aspirate fluid. P values assess ΔPGE_2 _(was the change from before to after treatment significant) and celecoxib level & ΔPGE_2 _(was the celecoxib level after treatment related to the change in PGE_2_)

2: Median change in PGE_2 _levels after treatment.

3: Only matched samples are included in this analysis, unlike the previous report which included PGE2 values for all subjects [16].

Celecoxib concentrations at the end of treatment were compared in pre- and postmenopausal women. Median concentrations in post- vs premenopausal women in the 200 mg group were: 267.5 ng/mL vs 117.6 ng/mL, and in the 400 mg group: 860.6 ng/mL vs. 227.3 ng/mL respectively (Table 2). Neither difference reached statistical significance (low dose, p = 0.26; high dose, p = 0.14).

### Celecoxib concentrations are associated with the decrease in PGE2 concentration in NAF but not plasma from high risk women taking high dose celecoxib

We compared plasma concentrations of celecoxib at the end of treatment with the change in NAF PGE_2 _from samples collected before and at the end of treatment. Celecoxib concentrations were significantly related (p = 0.006, r = -0.57) to the change in PGE_2 _in women taking 400 mg bid (Table 2) but not 200 mg bid celecoxib. The reason for high risk designation did not significantly influence a subject's PGE_2 _response to celecoxib.

Plasma celecoxib concentrations at the end of treatment were next compared to the change in PGE_2 _concentrations in plasma (Table 3). Unlike NAF, there was no association between celecoxib concentrations and the change in plasma PGE_2 _concentrations in high risk women taking 400 mg bid, nor in high risk women taking 200 mg bid.

**Table 3:** Median concentrations of PGE2 and celecoxib in plasma in ng/ml based on celecoxib dose^1^

**Population: Dose**		**Before (N)**	**After (N**	**After v**.	**P value**	
		**Treatment**	**Treatment**	**Before(N)**	**ΔPGE2**	**Celecoxib Level & ΔPGE_2_**
**200 mg twice daily**						
**Overall**	**PGE2**	0.24(20)	0.22(20)	-0.003(20)	0.70	0.25
	**Celecoxib**		253.4(20)			
**Premenopausal**	**PGE2**	0.42(6)	0.33(6)	0.008(6)	1.0	
	**Celecoxib**		195.3(6)			
**Postmenopausal**	**PGE2**	0.21(14)	0.20(14)	-0.004(14)	0.58	
	**Celecoxib**		267.5(14)			
**400 mg twice daily**						
**Overall**	**PGE2**	0.29(26)	0.21(26)	-0.016(20)	0.72	0.93
	**Celecoxib**		759.8(26)			
**Premenopausal**	**PGE2**	0.37(11)	0.37(11)	-0.0005(11)	0.77	
	**Celecoxib**		227.3(11)			
**Postmenopausal**	**PGE2**	0.28(15)	0.19(15)	-0.037	0.64	
	**Celecoxib**		860.6(15)			

1: N: sample size; NAF: nipple aspirate fluid. P values assess ΔPGE_2 _(was the change from before to after treatment significant) and celecoxib level & ΔPGE_2 _(was the celecoxib level after treatment related to the change in PGE_2_)

**Figure 1:**
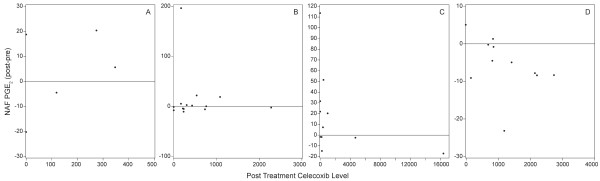
**Change in NAF PGE_2 _relative to plasma celecoxib level based on menopausal status and dose**. **(A) **Premenopausal women who received 200 mg celecoxib bid. **(B) **Postmenopausal women who received 200 mg celecoxib bid.**(C) **Premenopausal women who received 400 mg celecoxib bid. **(D) **Postmenopausal women who received 400 mg celecoxib bid. The horizontal line in each graph represents no change in PGE_2 _from pre- to posttreatment. Values above the horizontal line represent an increase in PGE_2 _posttreatment, values below a decrease.

At both the 200 mg and 400 mg doses of celecoxib, plasma concentrations of the drug trended lower in pre- than postmenopausal women. We therefore determined if there was a difference in the PGE_2 _response to celecoxib in NAF and/or plasma based on menopausal status (Figure 1). In the 400 mg group, the correlation coefficient was similar in both pre- (r = -0.52) and postmenopausal (r = -0.49) women (Figure 1C, D). Nonetheless, a significant decrease in NAF PGE_2 _was only observed in postmenopausal women (Table 2).

## Discussion

The objectives of the present study were to determine whether plasma celecoxib concentrations correlated with changes in either plasma or NAF PGE_2 _concentrations in women at increased risk for breast cancer, and whether a woman's pre- or postmenopausal status affected the plasma celecoxib concentration. This study extends our previous report that celecoxib treatment decreased PGE_2 _in NAF but not plasma in postmenopausal high risk women [16], by examining circulating celecoxib concentrations in matched plasma. In the current study, we confirmed our original observation that PGE_2 _levels in NAF, but not in plasma, decreased after celecoxib treatment in postmenopausal women, and that the PGE_2 _response in NAF correlated with plasma celecoxib concentration at the 400 mg bid dose level (figure 1). The significant decrease in NAF PGE_2 _that was observed in post- but not premenopausal women is likely because most premenopausal women had low (median 227 ng/ml) celecoxib levels, whereas most postmenopausal women had higher (median 860 ng/ml) levels.

Celecoxib concentrations were measured in plasma using two different dose regimens. Women taking 200 mg of celecoxib twice daily had a median plasma concentration of 253 ng/ml. When comparing the median plasma celecoxib concentration of pre- and postmenopausal women, a difference was observed (195 vs 267 ng/ml respectively). However, the difference was not statistically significant. This difference was also observed in the group of women taking 400 mg of celecoxib bid. Premenopausal women taking the higher celecoxib dose had a median plasma celecoxib concentration of 227 ng/ml while the postmenopausal women had a median value of 860 ng/ml. Again, this difference did not reach statistical significance, but is suggestive that there may be a relationship between menopausal status and plasma celecoxib concentration. Average time to collection after last dose was similar for both the premenopausal and postmenopausal subjects. It is possible, therefore, that the higher circulating concentrations of drug in postmenopausal women contributed to this greater effect. Although one report in which most of the enrolled subjects were male did not find an association of celecoxib level with age [19], it is possible that in women, menopausal status might influence the rate of clearance of the agent. The mechanisms of this possible difference are unclear but the qualitative observation warrants further investigation.

We are aware of six studies which evaluated steady state circulating celecoxib levels in humans (Table 4). Three were in healthy adults of various ages, one in a healthy elderly population, one in children with cancer, and one in adults with cancer. Sample sizes were generally small, with four of the six evaluating fewer than 10 subjects. Time on medication was one or two weeks in five of the studies. The dose ranged from 200 mg bid to 400 mg bid, with the pediatric dose adjusted per kg body weight to match an adult dose of 400 mg bid. Plasma concentrations ranged from 437 to 1087 ng/mL, although levels within individuals varied more than 100 fold [18]. Thus, the circulating celecoxib concentrations measured in our study are consistent with those previously reported in the literature.

**Table 4:** Studies measuring circulating celecoxib levels

**Population**	**Subjects**	**Weeks On Drug**	**Dose (mg)^1^**	**Last Dose (hrs)^2^**	**Source**	**Celecoxib concentration (ng/mL)**
Healthy Adults [18]	8	2	200 b.i.d.	up to 25	serum	437
Healthy Adults [18]	35	2	40 q.d.	13	plasma	568
Healthy Adults [18]	24	2	200 b.i.d.	up to 24	plasma	500
Healthy Adults [20]	35	6	400 q.d.	13	plasma	601
Elderly Adults [21]	8	1	200 b.i.d.	2	plasma	755
Pediatric cancer patients [22]	9	250 mg/kg	b.i.d.	6	plasma	668
Adult cancer patients [23]	4	week^3^	400 b.i.d.	6–8	plasma	1087

1: b.i.d.: twice daily; q.d.: once daily

2: Unless otherwise stated, average time from last dose celecoxib to blood draw

3: The text did not define how many weeks the subjects took the medication

In three of the groups of women (premenopausal @ 200 mg bid, postmenopausal @ 200 mg bid and premenopausal @ 400 mg bid), plasma celecoxib concentrations ranged between 195 and 267 ng/ml. In contrast, the postmenopausal women demonstrated a median plasma celecoxib concentration of 860 ng/mL. There was a strong inverse correlation both in pre- and postmenopausal women receiving 400 mg bid celecoxib between plasma celecoxib concentrations and naf PGE_2_. It therefore appears that, regardless of menopausal status, it is the circulating level of celecoxib that is important, with low levels having little influence on PGE_2_, and higher doses decreasing PGE_2_. Although we cannot exclude the possibility that low celecoxib levels increase PGE_2_, changes in PGE PGE_2 _at lower celecoxib levels were not significant.

The lack of effect of celecoxib at either 200 or 400 mg twice daily on the plasma PGE_2 _concentration is not unexpected. Celecoxib is a specific COX-2 inhibitor and its clinical advantage is that it does not inhibit COX-1. COX-1 is assumed to be a constitutively expressed enzyme that is present in almost every cell of the body. COX-2 is assumed to be an inducible enzyme that responds to specific conditions and environments. Since PGE_2 _is a local mediator, most of the circulating plasma PGE_2 _probably represents COX-1 activity. Since celecoxib specifically inhibits COX-2, the PGE_2 _present in NAF is likely the product of both COX-1 and COX-2 activity, with the downregulation of PGE_2 _reflecting the action of celecoxib. We suspect that the significant decrease (from a median of 8.33 to 6.81 ng/ml) which occurred in the group with the highest overall celecoxib levels,. Is due to the effect of celecoxib on PGE_2 _contributed by COX-2, but not that contributed by COX-1, which is why levels decreased a median of 18% rather than to a greater extent.

We observed differences in the overall median plasma concentration of celecoxib in women receiving a dose of 200 mg bid (224 ng/ml) versus 400 mg bid.(760 ng/ml). Although no statistically significant differences were seen in the plasma concentration of celecoxib in pre- versus postmenopausal women in either dose group, there was a qualitative trend for higher plasma concentrations in the postmenopausal group. We also observed a significant decrease in NAF PGE_2 _among women taking 400 mg celecoxib bid for 2 weeks where the median circulating dose of celecoxib was 860.6 ng/mL, but not in women taking 200 mg bid, where the median circulating dose was less than 300 ng/mL. An observational study observed that long term (at least 2 years) use of low dose (200 mg daily) celecoxib significantly decreased breast cancer risk [7]. Our data suggest that in the short term 200 mg bid is not sufficient to reliably inhibit breast tissue formation of PGE_2_, although long-term therapy may.

Celecoxib therapy is associated with cardiovascular risk and its value as a chemopreventative agent may be called into question. However, the currently approved breast cancer chemopreventive agents tamoxifen and raloxifene have side effects of hot flashes, vaginal discharge, blood clots and stroke. Tamoxifen also increases the risk of endometrial carcinoma, endometrial sarcoma and cataracts. Aromatase inhibitors, which are under investigation as breast cancer chemopreventive agents, increase the risk of osteoporosis. If celecoxib is to ever be used as a chemopreventive agent, there is a need to balance breast cancer risk reduction while minimizing risk of cardiovascular toxicity, which has only been associated with high dose celecoxib. It is important to determine an optimal celecoxib dose which minimizes toxicity while conferring a cancer protective effect. Under these conditions, celecoxib may prove to be a valuable chemopreventive agent.

In conclusion, our findings suggest that monitoring plasma celecoxib concentrations may provide a method to determine response to a an intermediate marker of breast cancer. Long term studies are needed to assess if plasma celecoxib concentrations will predict the breast cancer preventive effect of the agent.

## Conclusion

In this short term study, plasma concentrations of celecoxib correlated with downregulation of PGE_2 _production by breast tissue in women taking 400 mg bid, but not the 200 mg bid dose. Given epidemiologic studies in breast cancer suggesting a chemopreventive effect of lower doses after longer term use, prospective studies using lower doses, as well as chemoprevention strategies synergistic with celecoxib to downregulate PGE_2 _are of interest, in order to minimize the celecoxib dose required to have an effect.

## Abbreviations

COX: cyclooxygenase; nipple aspirate fluid: NAF; PG: prostaglandin

## Competing interests

The author(s) declare that they have no competing interests.

## Authors' contributions

ERS designed the study, enrolled subjects, and performed the majority of manuscript preparation. WQ conducted all PGE_2 _analyses. RLR and JTF assisted with manuscript preparation and critique. JEH performed the statistical analyses, GR and YCC conducted the celecoxib analyses. All authors read and approved the final manuscript.

## Pre-publication history

The pre-publication history for this paper can be accessed here:


